# Characterization of DNA methylation in PBMCs and donor-matched iPSCs shows age-related methylation is reset during stem cell reprogramming

**DOI:** 10.1371/journal.pone.0356501

**Published:** 2026-09-08

**Authors:** Xylena Reed, Cory A. Weller, Sara Saez-Atienzar, Alexandra Beilina, Sultana Solaiman, Makayla Portley, Mary Kaileh, Roshni Roy, Jinhui Ding, A. Zenobia Moore, D. Thad Whitaker, Bryan J. Traynor, Luigi Ferrucci, J. Raphael Gibbs, Sonja W. Scholz, Mark R. Cookson

**Affiliations:** 1 Cell Biology and Gene Expression Section, Laboratory of Neurogenetics, National Institute on Aging, National Institutes of Health, Bethesda, Maryland, United States of America; 2 Center for Alzheimer’s and Related Dementias, National Institute on Aging and National Institute of Neurological Disorders and Stroke, National Institutes of Health, Bethesda, Maryland, United States of America; 3 DataTecnica LLC, Washington, District of Columbia, United States of America; 4 Neuromuscular Disease Research Section, Laboratory of Neurogenetics, National Institute on Aging, National Institutes of Health, Bethesda, Maryland, United States of America; 5 Neurodegenerative Diseases Research Section, National Institute of Neurological Disorders and Stroke, Bethesda, Maryland, United States of America; 6 Laboratory of Molecular Biology and Immunology, National Institute on Aging, National Institutes of Health, Baltimore, Maryland, United States of America; 7 Computational Biology Group, Laboratory of Neurogenetics, National Institute on Aging, National Institutes of Health, Bethesda, Maryland, United States of America; 8 Translational Gerontology Branch, National Institute on Aging, National Institutes of Health, Baltimore, Maryland, United States of America; 9 Department of Neurology, Johns Hopkins University Medical Center, Baltimore, Maryland, United States of America; University of Newcastle, UNITED KINGDOM OF GREAT BRITAIN AND NORTHERN IRELAND

## Abstract

DNA methylation is an important epigenetic mechanism that helps define and maintain cellular functions. It is influenced by many factors, including environmental exposures, genotype, cell type, sex, and aging. Since age is the primary risk factor for developing neurodegenerative diseases, it is important to determine if age-related DNA methylation is retained when cells are reprogrammed to an induced Pluripotent Stem Cell (iPSC) state. Here, we selected peripheral blood mononuclear cells (PBMCs; n = 99) from a cohort of diverse and healthy individuals enrolled in the Genetic and Epigenetic Signatures of Translational Aging Laboratory Testing (GESTALT) study to reprogram to iPSCs. After reprogramming, the resulting iPSCs were evaluated for DNA methylation signatures to determine if they reflect the confounding factors of aging and environmental effects. Data from genome-wide DNA methylation arrays in both cell types showed that age-related methylation measured by epigenetic clocks is largely reset to an early methylation age after reprogramming of PBMCs to iPSCs. We further examined the epigenetic age of each cell type using an Epigenome-wide Association Study (EWAS) and identified a set of methylation Quantitative Trait Loci in each cell type. Our results show that age-related DNA methylation is largely reset in iPSCs, and each cell type has a unique set of methylation sites that are modified by population-level genetic variation.

## Introduction

Methylation of cytosine residues is a stable epigenetic modification of DNA seen across species and is responsible for controlling many aspects of gene expression [1]. In mammalian cells, cytosine is methylated predominantly at CpG sites by the enzyme DNA methyltransferase 1. While many CpG sites in the genome show high levels of constitutive methylation, the proportion of DNA methylated at other sites depends on the cellular differentiation state and other environmental and biological effects, including aging [2–4]. DNA methylation also correlates with genetic variation, leading to the establishment of methylation quantitative trait loci (methQTL), similar to expression quantitative trait loci (eQTL) for RNA expression [5–8]. Thus, the overall proportion of CpG methylation in each tissue reflects a complex interplay between genetic and non-genetic influences.

Induced pluripotent stem cells (iPSC) are widely used to model cell biological events without cell transformation. For example, iPSC models have been used to replicate tissue-based methQTL and eQTL in an endogenous genomic context, suggesting that these models retain genetically encoded influences on epigenetics and gene expression [9,10]. However, there has been some disagreement on whether iPSC models retain epigenetic marks that arise from cellular differentiation or organismal aging, as these are rewritten during reprogramming [11–13]. While some types of DNA methylation may be lost, iPSCs can be grown under consistent conditions and so, by inference, the contribution of environmental factors should be consistent between different donor lines. Thus, iPSC models may be useful in identifying methQTL independent of other sources of methylation variation [14].

Here, we generated a new collection of iPSC lines from a cohort of healthy donors, ranging from 22 to 92 years old, to examine the effects of cellular reprogramming on CpG methylation. We first obtained peripheral blood mononuclear cells (PBMCs) collected by apheresis from 99 individuals in the ongoing Genetic and Epigenetic Signatures of Translational Aging (GESTALT) longitudinal study that aims to identify biomarkers related to aging [15–19]. The strict inclusion criteria for the study’s participants result in the retention of healthy individuals, allowing us to examine CpG methylation signatures in the absence of diagnosed disease. Additionally, the availability of genotyping and methylation data from the same donors before and after reprogramming allowed us to compare methQTL between matched iPSCs and PBMCs.

We show that iPSCs lack age-related methylation signatures tested using five different epigenetic clocks and an Epigenome-wide Association Study (EWAS). Additionally, CpG methylation patterns are highly distinct between iPSCs and PBMCs, and while we recovered abundant evidence of genetic effects on CpG methylation in both PBMCs and iPSCs, some of which were correlated between cell types, we found that there is a unique set of methQTL for each cell type. We found that methQTL in PBMCs are enriched for gene associations that are involved in biological processes involving lipid localization and steroid secretion.

## Materials and methods

### Cohort selection

PBMCs were banked from the Genetic and Epigenetic Signatures of Translational Aging Laboratory Testing Study (GESTALT) participants (n = 99) where the minimum age for enrollment was 20 years. As previously described [15–18], GESTALT participants were clinically healthy at the time of enrollment based on eligibility criteria, they were not on medications except of one antihypertensive drug, free of major diseases except of history of silent cancer for more than 10 years. Participants had no physical or cognitive impairments, non-smokers and had a body mass index ≤ 30 kg/m2. Full study inclusion criteria can be found at https://clinicaltrials.gov/study/NCT02339012#participation-criteria. Human subjects research for the GESTALT protocol (initial approval T-AG-0021, current study number 15-AG-0063) was approved by the Institutional Review Board of the National Institutes of Health, and all participants provided written informed consent at each visit. PBMCs were accessed for this study between July 1, 2019 and October 31, 2019. All participants have been de-identified and were not identifiable to authors after collection.

### PBMC isolation

Peripheral blood mononuclear cells (PBMCs) were isolated from cytapheresis packs of the GESTALT donors by density gradient centrifugation using Ficoll-Paque Plus (Cytiva, Marlborough, MA). After subsequent washing steps with DPBS (Quality Biological, Gaithersburg, MD), PBMCs were cryopreserved as 10 million cells per ml and stored in a liquid nitrogen freezer until used.

### PBMC DNA extraction and genotyping

DNA was extracted from 1–2 million PBMCs using either DNAQuik DNA extraction protocol (Reprocell, Beltsville, MD) or Qiagen DNeasy kit (Genetic Resources Core Facility, JHU, Baltimore, MD). 1 µg of DNA was sent to Novogene (Chula Vista, CA) for genotyping.

### Reprogramming

PBMCs were sent to Thermo Scientific (Carlsbad, CA) for reprogramming. First, the cells were tested for mycoplasma, using the MycoSEQ^TM^ kit (Applied Biosystems, 4460623). They were also confirmed to be free of human pathogens: hepatitis B virus (HBV), hepatitis C virus (HCV), Human Immunodeficiency Virus type 1 (HIV-1), Human Immunodeficiency Virus type 2 (HIV-2), Human T-lymphotropic virus type 1 (HTLV) I/II, Cytomegalovirus (CVM), Epstein–Barr (EBV), Herpes simplex virus 1 (HSV-1), and Herpes simplex virus 2 (HSV-2). If any lines tested positive for any of these, they were excluded from reprogramming. Mycoplasma and human pathogen-negative PBMCs were thawed, assessed for viability, and transduced with CytoTune^TM^ reprogramming particles from the Cytotune^TM^-iPS 2.0 Sendai Reprogramming kit (Invitrogen, A16518). Three days after transduction, the cells were re-plated onto Geltrex^TM^ (LDEV-Free hESC-qualified Reduced Growth Factor Basement Membrane Matrix)-coated dishes (Gibco, A1413202). The cells were maintained in Essential 8^TM^ medium (Gibco, A1517001), which was changed daily until colonies were picked for further analysis.

Four to five weeks after introducing Cytotune^TM^ 2.0 particles, pluripotent stem cell-like colonies emerged and were picked based on morphological characteristics. Colonies were cut out using a needle and plated into a well of Geltrex^TM^-coated 24-well plates containing Essential 8^TM^ Medium. Cloneses were expanded, and then dissociated using TrypLE^TM^ Select Enzyme (Gibco, 12605010) and single cell passaged into one well of a Geltrex^TM^-coated 12-well plate containing StemFlex^TM^ Medium (Gibco, A3349401), supplemented with RevitaCell^TM^. Once established in 12-well plates, the two best-looking colonies were dissociated using Versene Solution (Gibco, 15040066) and plated in Geltrex^TM^-coated 6-well plates. The cell lines were then expanded and banked for liquid nitrogen storage using the PSC Cryopreservation kit (Gibco, A2644601). Upon expansion, cell pellets were collected for Mycoplasma testing, TaqMan® iPSC Scorecard^TM^ (ThermoFisher, A15872), Pluritest^TM^ (Thermo Scientific, A38154), and Karyostat+^TM^ (Thermo Scientific, A52986). Two clones for each donor were banked and defined as A and B.

### Culture of iPSCs

All resulting iPSC lines were expanded in Essential 8^TM^ Flex medium (Gibco, A2858501) on Matrigel (Corning, 354230)-coated dishes. RevitaCell (Gibco, A2644501) supplement was added to the medium for one day after thawing and cell splitting. iPSCs were passaged using TrypLE^TM^ Select Enzyme or Cell Dissociation Buffer (Gibco, 13151014) for a total of 1–2 passages and recovered post-split using Essential 8^TM^ Flex Medium with 1X RevitaCell^TM^. Expanded iPSCs were frozen for storage using Synth-a-Freeze^TM^ Cryopreservation Medium (Gibco, A1254201).

### DNA isolation and genotyping iPSCs

Approximately 1x10^6^ cells were pelleted and frozen for DNA isolation from each clone that was successfully expanded. Genomic DNA was extracted from iPSCs using the Maxwell RSC Tissue DNA kit (Promega, AS1610) following the manufacturer’s instructions. DNA samples were quantified, and 250 ng of genomic DNA were genotyped for each sample using the Neuro Global Diversity Array-8 v.1 LCG with Neuro Booster content (Illumina, 20031815 and 20042459) [20] according to the manufacturer’s instructions. Briefly, genomic DNA was amplified and enzymatically fragmented. Following alcohol precipitation, the resulting fragments were resuspended and hybridized to the array. Automated allele-specific, enzymatic base extension and fluorophore staining were performed using a liquid-handling robot (Tecan, Research Triangle Park, NC). The stained arrays were washed, sealed, and vacuum-dried before scanning on the Illumina iScan instrument.

### Genotyping quality control and imputation for iPSCs

Raw data files were imported into the GenomeStudio Genotyping Module (v.2.0, Illumina) using a custom-generated sample sheet, and genotypes were called using a GenCall threshold of 0.15. The genotype data were exported in a ped-file format for downstream quality control. Genotype quality control was performed following standard criteria. Samples meeting the following criteria were excluded: 1) samples with inbreeding coefficient F-statistic (estimated by PLINK) outside of the range (−0.15, 0.15); 2) call rate ≤ 95%; 3) mismatch between reported sex and genotypic sex; or 4) duplicate samples indicated by pi-hat statistics > 0.8. Ancestry was ascertained based on principal component analysis compared to the HapMap 3 Genome Reference Panel. No samples were excluded from the follow-up analysis based on ancestry. Related samples were also included for imputation (defined as having a pi-hat > 0.125). Variants-wise quality was also applied, and variants were removed if they were present in any of the following categories: 1) monomorphic SNPs; 2) palindromic SNPs; 3) variants with haplotype-based nonrandom missingness (*p* ≤ 1.0 × 10^−4^); 4) variants with an overall missingness rate of ≥ 5.0%; 5) non-autosomal variants; and 6) variants that deviated from Hardy-Weinberg equilibrium (*p* ≤ 1.0 × 10^−10^). Imputation was performed against the Trans-Omics for Precision Medicine (TopMed) imputation reference panel for hg38 via minimac4 using data phased by Eagle v2.4 provided by TopMed Imputation Server [21]. After imputation, only variants with an imputation quality score R^2^ > 0.8 and minor allele frequency (maf) > 0.01 were included in the analysis.

### DNA methylation profiling

For each clone that was successfully expanded, 450 ng of genomic DNA was bisulfite converted (Zymo Research, D5003), hybridized onto Infinium HumanMethylation EPIC BeadChips v1.0 (Illumina, 2016168), and stained according to the manufacturer’s protocol. Briefly, after DNA amplification at 37°C for 20 hours, the samples were enzymatically fragmented and precipitated, followed by DNA hybridization and automated Beadchip staining using a liquid handling robot (Tecan). Methylation arrays were washed, sealed, and vacuum-dried before scanning using the iScan instrument (Illumina).

### Methylation quality control and normalization

Methylation data quality control (QC) was performed in two rounds using the meffil R package (version 1.3.3) [22]. The first round identified low quality samples based on the following criteria: 1) samples with a predicted median methylated signal > 3 standard deviations (SD) from the expected, 2) samples that had > 10% of probes with bead numbers < 3, 3) samples that had > 10% of probes with detection *p*-value > 0.01, 4) samples with gender mismatch between the reported and predicted gender (sex outlier value > 5 SD from the mean), 5) samples with a genotype concordance < 80% (genotype mismatch). Methylation was measured in all available clones, but only one clone was used for downstream analyses. Clone A was selected if available and passed all QC measures, otherwise clone B was used. Using only one clone allowed for a better matched comparison with the single PBMC sample from each donor. After removing samples that failed QC, a second round of filters identified low-quality probes for exclusion. CpGs within single nucleotide polymorphisms with maf > 1% located within five nucleotides of the target sites, probes tagging non-unique 3΄-subsequences of 30 or more bases long, and cross-reactive probes as previously described [23] were all excluded from the analysis. Additional EPIC-specific cross-reactive probes were obtained from the Maxprobes R package (version 0.0.2) and removed [24,25]. Principal component analysis was performed on the control matrix and the 20,000 most variable probes, and the top ten principal components, in addition to the EPIC array slide as a random effect, were used as covariates for quantile normalization (meffil.normalize.quantiles).

### Epigenetic clock analysis

The calculation of the epigenetic age using the Horvath [11], Hannum’s [26], phenoAge [27], BLUP (Best Linear Unbiased Prediction) [28], and EN (Elastic Net) clocks were performed with the Methylclock R package (version 1.6.0) [29].

### Analysis of absolute telomere length

The average telomere length for each reprogrammed iPSC line was determined using the Absolute Human Telomere Length Quantification qPCR Assay Kit (Sciencell, 8918). Briefly, 5 ng of DNA was used for each reaction with Single Copy Reference (SCR) primers or Telomere (TEL) primers. Each iPSC line was measured in technical triplicate for both primer pairs. A reference human genomic DNA sample of known length (935 ± 45 kb per diploid cell; Sciencell, 8918b, Lot #33114) was included in triplicate with both primer pairs. The relative telomere length and total telomere length of each iPSC line was determined as the protocol directed using the ^∆∆^Cq method [30]. The average telomere length on each chromosome end (telomere length per diploid cell divided by 92 chromosome ends in one diploid cell) was plotted against the age of the donor when PBMCs were isolated for reprogramming. Correlation between donor age and average telomere length identified the Pearson r = −0.0805 (p-value = 0.4612).

### PBMC cell type predictions

To account for cell type heterogeneity within PBMC samples, we performed EWAS and methQTL with covariates of cell type proportions. To generate covariates, we used the R package Meffil’s meffil.estimate.cell.counts.from.betas function [22]. Using published sorted Cord Blood Mononuclear cells [31] as a model, the function predicts cell type proportion for seven cell types (B, CD4T, CD8T, granulocytes, monocytes, natural killer lymphocytes, and nucleated red blood cells) based on methylation beta values for each sample.

### Epigenome-wide association study

Epigenetic genome-wide association analyses were performed independently in PBMCs and iPSCs using the *meffil.ewas* function of the R package meffil (v 1.3.3) [22]. Linear regression was performed at each site (n = 748,760 probes in the PBMC dataset and n = 732,148 probes in the iPSC dataset) testing for an association between DNA methylation β values and the donor’s age at the time of the sample collection (age at collection). For covariates, the model included the sex, first five principal components from LD-pruned genotypes [32], and the first five principal components from the top 20,000 most variable methylation probes. For PBMCs, analysis was also repeated with cell type proportion predictions as covariates. We considered significant EWAS loci as those with a p-value of less than a genome-wide threshold of 5 × 10^−8^. Probes were mapped to gene names using the EPICv2 hg38 annotation manifest.

### methQTL analysis

Genome-wide SNP-methylation probe pairs were tested using TensorQTL, a GPU-enabled QTL mapper that achieves >200 fold faster *cis-*QTL calculation than the CPU-based mappers [33]. Briefly, an additive linear model tested if genotypes within 1MB of a probe site (*i.e.,* the number of alternative alleles) predicted DNA methylation. iPSC and PBMC methylation datasets were processed independently and included for covariates sex, age, and the first ten principal components from LD-pruned genotypes. For PBMCs, analysis was also repeated with cell type proportion predictions as covariates. Significant methQTL were defined as those with an FDR-adjusted p-value (q-value) of less than 0.05.

### Gene ontology enrichment analysis

MethQTL from the previous analysis were filtered to datasets that were unique to each cell type by removing any overlapping sites. Enrichment was calculated using the enrichGO and enrichKEGG functions from R *clusterProfiler* v4.10.1 [34,35], correcting for multiple testing using the BH method.

### Imprinting gene analysis

We included a list of known human imprinting genes from https://www.geneimprint.com.

## Results

### Generation of a population resource for iPSC from diverse, healthy donors across the age span

**Fig 1:**
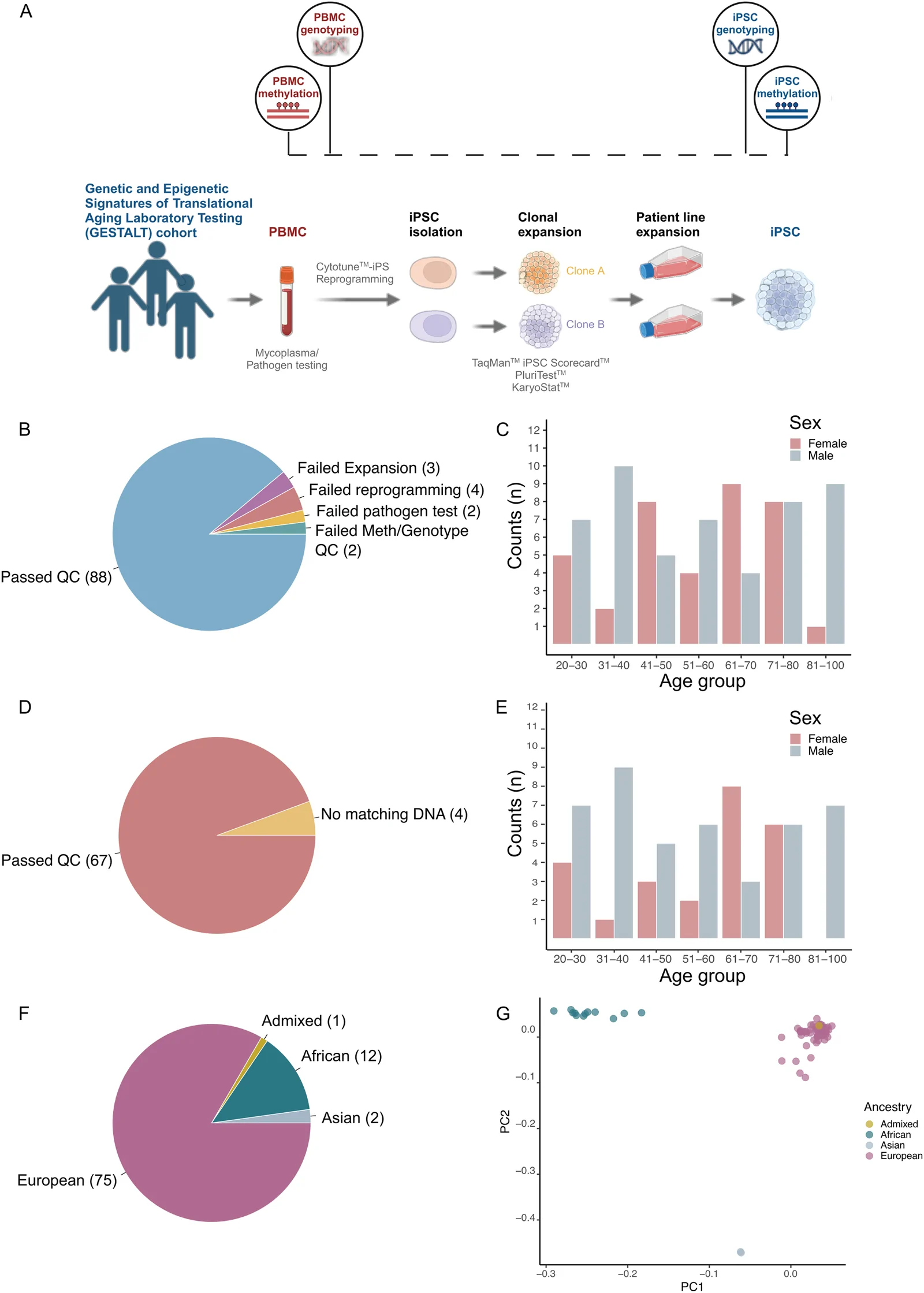
Experimental workflow and sample characteristics in a healthy donor series. (A) Experimental workflow. (B) Number of iPSC lines passing all quality control thresholds, and those that failed for each step at reprogramming, expansion, pathogen testing or methylation or genotype quality control checks. (C) Count of iPSC lines passing QC binned by age and sex. (D) Number of PBMC donor methylation samples passing data quality thresholds, and those that did not have donor matched iPSC DNA. (E) PBMC samples that passed QC checks binned by age and sex. (F) Number of samples split by ancestry group after QC. (G) Genetic principal components analysis (PCA) of remaining samples compared to the HapMap 3 Genome Reference Panel. This figure was created with the help of biorender (panel A).

We first set out to generate a series of iPSC lines from PBMCs collected from 99 individuals in the GESTALT cohort [15–19] (Table 1 and S1 Table). The GESTALT study donors ranged in age from 20 to 100 years old at time of collection, had no diagnosed diseases, and were cognitively and physically unimpaired. While we cannot rule out any undiagnosed conditions, these participants were deeply clinically phenotyped at each sequential visit and appeared to be representative of a healthy collection of donors across the age-span. Our overall goal was to address the relative contributions of aging, environment, genetics and cell type on DNA methylation by comparing PBMC methylation patterns to those in cells reprogrammed to iPSCs (Fig 1A). After testing PBMCs for mycoplasma and human pathogens, cells were reprogrammed using Cytotune delivered by Sendai virus, with two clones retained for each donor. Of the initial 99 donors, 93 lines were effectively reprogrammed (S1A Fig). Quality of reprogramming was evaluated using a TaqMan-based iPSC Scorecard [36], PluriTest, and KaryoStat (S1B Fig and S2 Table), and all lines passed conventional thresholds for each test.

**Table 1:** Demographics of iPSC and PBMC lines that passed QC.

		iPSC	PBMC (%)
**Sex**	**Female**	39	24
	**Male**	51	43
**Age at collection, mean (SD)**		55.17 (±19.47)	54.37 (±20.03)
**Ancestry**	**Admixed**	1	8
	**African**	8	7
	**Asian**	2	2
	**European**	75	50
**Total**		90	67

Count of samples in remaining in each category after QC filters, split by iPSC and PBMC. Mean age at collection and standard deviation.

Of the 93 reprogrammed cell lines, 90 were successfully expanded. Following clone expansion, each line was genotyped on an Infinium Global Diversity Array. We confirmed the identity of each line by comparing iPSC genotypes to genotypes previously generated from the PBMCs of each donor, and two lines were discarded for failing methylation and genotyping. After quality control analysis, the iPSC cohort consisted of 88 total lines made up of individuals of European (n = 75), Black or African American (n = 12), and Asian ancestries (n = 2), as well as one individual who self-reported multiple ethnicities (based on genetic PCs this individual maps with the European samples in Fig 1G) (Fig 1 F and G; Table 1, S1 Table). The samples that remained after all reprogramming and QC steps ranged in age from 22 to 92 years at the time of PBMC collection. Thus, we successfully generated a cohort of 88 iPSC lines, with both genetic and DNA methylation data, from donors across the age-span and multiple genetic ancestries.

### Cell state and inter-individual differences contribute to methylation status

We generated genome-wide methylation data from both clones for each iPSC line using the Illumina Infinium MethylationEPIC v2.0 array and performed stepwise quality control checks at sample- and probe-level after normalization (Methods). Methylation data from the EPIC array were also available from a subset of the original PBMC samples (n = 73). CpG methylation values showed a bimodal distribution for both PBMCs and iPSCs, as expected. After quality control, we retained 67 donor-matched iPSC-PBMC pairs and compared the resulting methylation signatures from the same donors across iPSC clones and between cell types (Fig 1D and E; Table 1). We calculated the Pearson correlation coefficient between samples using methylation probes that were informative for both cell types, i.e., probes within the top 5000 most variable probes for both iPSCs and PBMCs. We found that the iPSC replicate clone-to-clone correlation (ρ = 0.911 ± 0.057, number of samples with two clones = 70) was higher than the PBMC-to-iPSC correlation within the same donor (ρ = 0.714 ± 0.039, Table 2, S2 Fig). Both measures were significantly higher than the mean correlation between iPSCs from different donors (ρ = 0.522 ± 0.0343). All group comparisons were significantly different from each other (ANOVA: df = 4, *F* = 10320, *p* < 2 × 10^−16^; Tukey HSD, all comparisons: p-value < 2 × 10^−16^). Based on the high correlation across clones we selected only one iPSC clone for downstream analyses, prioritizing clone A when it was available, to pair with the single PBMC measure from each donor. We also performed a correlation analysis of DNA methylation against all PBMCs-iPSC clones for cells from different donors and saw a much lower correlation (ρ = 0.383 ± 0.055; S3 Fig). Collectively, these data show that inter-individual differences contribute to methylation status, but also that a significant source of variability for DNA CpG methylation relates to cell state.

**Table 2:** Methylation correlation between and within donors and cell types.

Comparison	Cells	Number of comparisons	Mean	SD
Same Donor	iPSC to iPSC	70	0.911	0.056
Same Donor	iPSC to PBMC	128	0.714	0.039
Different Donor	iPSC to iPSC	12491	0.522	0.073
Different Donor	PBMC to PBMC	2485	0.578	0.050
Different Donor	iPSC to PBMC	11161	0.383	0.055

### iPSC clones reset age-associated DNA methylation

**Fig 2:**
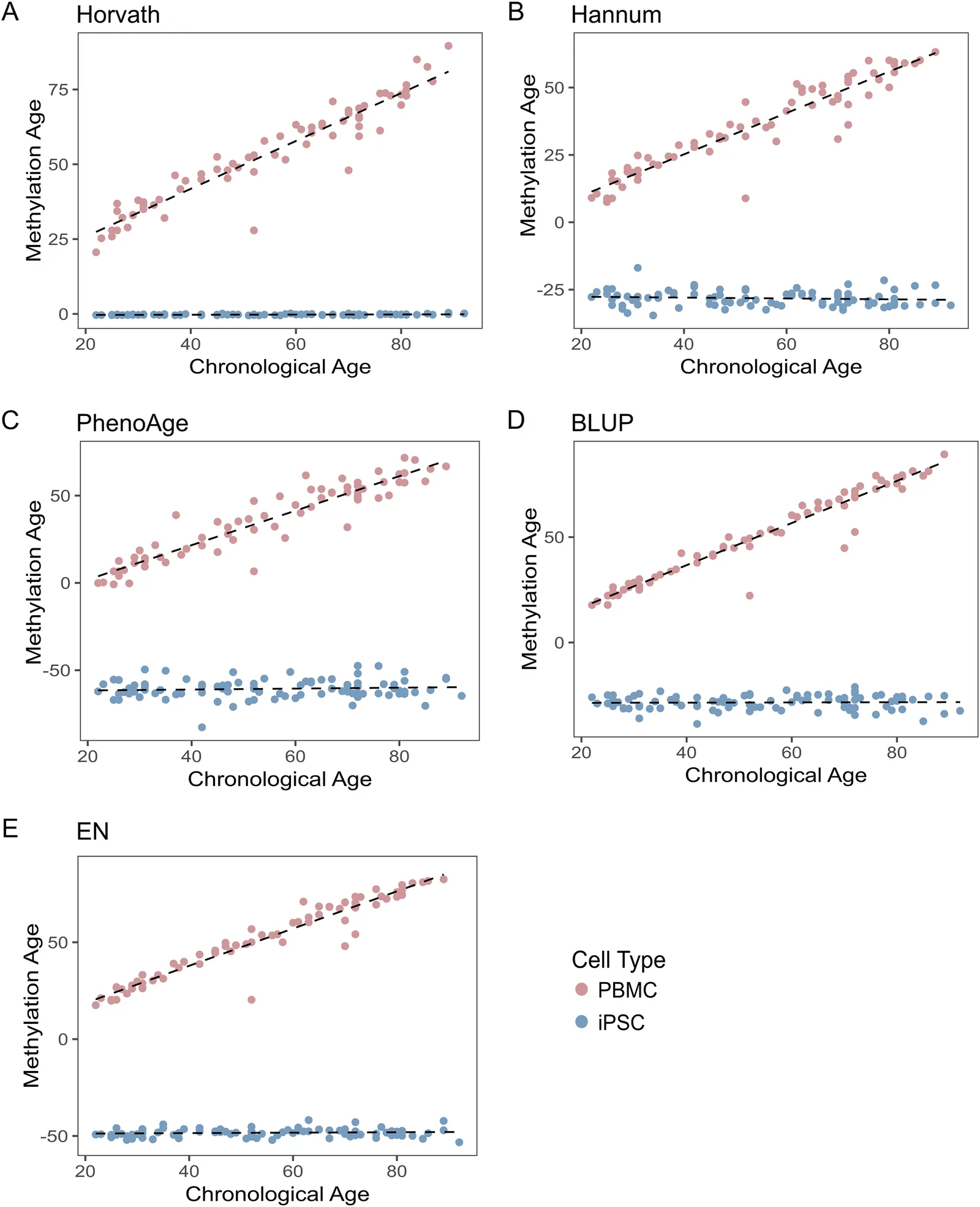
Comparison of methylation aging signatures in PBMCs and iPSCs across multiple epigenetic clocks. (A) Using the Horvath epigenetic clock, the correlation values between chronological age (x-axis) and methylation age (y-axis) in PBMCs (red points) and iPSCs (blue points. (B) Hannum clock (C) PhenoAge clock (D) Best Linear Unbiased Prediction (BLUP) clock and (E) Elastic Net (EN) clock.

We next evaluated whether iPSC lines are epigenetically young relative to their donors in this healthy population study across the lifespan. We estimated the epigenetic age using the Horvath multi-tissue predictor of epigenetic age, which considered the methylation state of 353 CpGs [11]. As expected, PBMC DNA methylation was strongly correlated with donor age (r = 0.905, p-value = 2.95 x 10^−37^, n = 67; Fig 2A). The estimated average methylation age was 53.33 ± 16.75 years. In contrast, iPSC DNA methylation showed no correlation with donor age (r = 0.09, p = 2.24 x 10^−3^, n = 70), and the estimated methylation average age was −0.24 ± 0.17 years. These results were confirmed with additional DNA methylation clocks, including the clock developed by Hannum et al. (Fig 2B), the phenoAge clock (Fig 2C), and two age predictors described by Zhang et al. based on best linear unbiased prediction (BLUP; Fig 2D), and elastic net prediction (EN; Fig 2E) [26–28]. In all analyses, the epigenetic clocks showed no correlation between methylation age and chronological age in iPSCs, while the corresponding donor PBMCs displayed a strong correlation, indicating that the loss of age-associated DNA methylation during reprogramming was robust across different measures (S3 Table).

**Fig 3:**
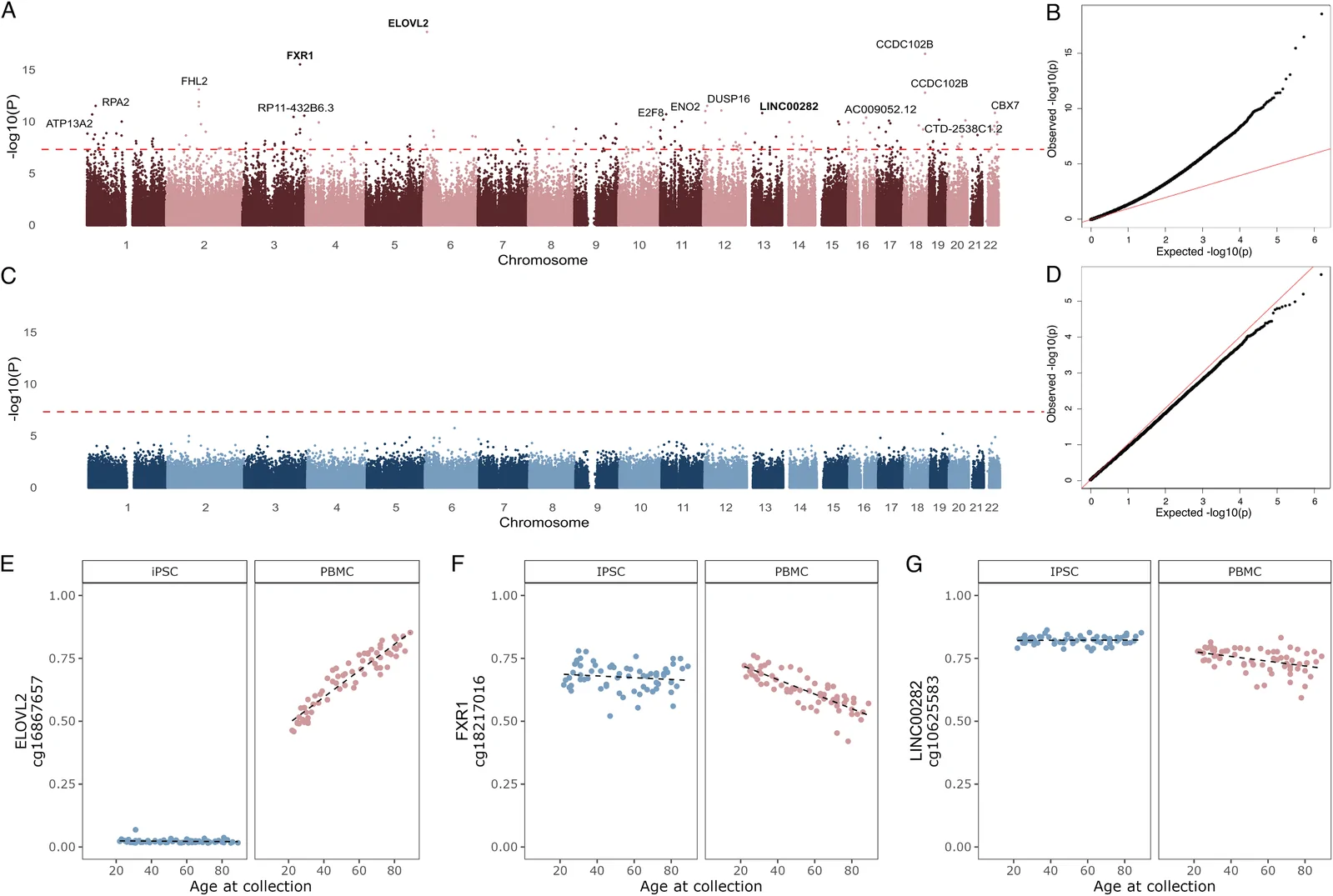
Epigenome-wide Association Study (EWAS) in PBMCs and reprogrammed iPSCs. (A) Manhattan plot showing associations between DNA methylation at common CpG sites and age in PBMCs. The dashed red line shows genome-wide significant associates above p-value < 5x10^−8^ (B) The association analysis quantile-quantile (QQ) plots corresponding to PBMCs. Expected p-values are on the x-axis, and the observed p-values are on the y-axis. P-values are transformed to a -log10 scale. (C) Manhattan of common CpG sites and age in iPSCs. (D) QQ plot corresponding to the iPSC EWAS. (E) Age at collection (x-axis) vs CpG methylation (y-axis) plotted by sample at cg16867657, near *ELOVL2*, in iPSC (blue) and PBMC (red). (F) Age vs CpG methylation at cg18217016, near *FXR1*, in iPSC and PBMC. (G) Age vs CpG methylation at cg10625583, near *LINC00282*, in iPSC and PBMC.

DNA methylation clocks are based on multiple regression models across various informative genomic sites. As a result, we hypothesized there might be residual aging signals outside of the tested groups of CpG methylation sites. We therefore considered whether other individual DNA CpG methylation sites may show an association with donor age and performed an Epigenome-Wide Association Study (EWAS) using donor age as an outcome measure in PBMCs and iPSCs. We identified age-associated changes in methylation in PBMCs yet recovered no significant associations within the iPSC data (Fig 3A-D). For PBMCs, while we replicated some sites already associated with aging by one or more methylation clocks (for example the most significant site overall, cg1687657 near *ELOVL2* (Fig 3E), we also identified new sites not previously associated with aging. For example, two newly identified sites, cg18217016 (near *FXR1*) and cg10625583 (near *LINC00282*), showed an age-associated decrease in methylation in the PBMC EWAS and have not previously been reported to be associated with aging (Fig 3 F and G). Since PBMCs are made up of multiple cell types and the proportions can vary from sample to sample we used Meffil to estimate the proportions of cell types within each sample (S4 and included the cell type proportions (S4 Table) as covariates in a second EWAS calculation (S4 Fig). Cell type proportions were similar across samples with most cells being CD4 + T cells (0.31±0.063), CD8 + T cells (0.23±0.083) and monocytes (0.21±0.074). There were smaller proportions of B cells (0.13±0.044), natural killer cells (0.11±0.064) and red blood cells (0.016± 0.011). Only one sample had an identifiable proportion of granulocytes. After cell type correction, signals near *FXR1* and *ELOVL2* remained significant, however the association between cg10625583 (near *LINC00282*) and age was no longer significant. The total number of significant sites identified was reduced, but it did not greatly affect the overall outcome of the PBMC EWAS. These results demonstrate that iPSCs do not retain age-related methylation signatures, even outside of the sites used in epigenetic clocks. Additionally, EWAS may be used, even in a small sample size, to identify factors associated with aging in both purified iPSCs and mixed PBMCs.

To provide an independent method for evaluating cellular age, we measured telomere length in all iPSCs. We found that telomere length was not correlated with donor age (Pearson r = −0.0805, p-value = 0.4612) and appeared to be reset similarly to DNA methylation (S5 Fig). This is consistent with previous reports in mouse Embryonic Stem Cells and human iPSCs which have shown that Telomerase Reverse Transcriptase (*TERT*), the enzyme responsible for maintaining telomere length, is highly expressed in stem cells relative to somatic cells and telomere length is positively correlated with pluripotency [37,38]. Collectively, these data confirm that our iPSCs are epigenetically reprogrammed to a young state, in contrast to PBMCs from the same healthy donors across the human age span.

### methQTL in PBMCs and iPSCs are distinguished by cell state

**Fig 4:**
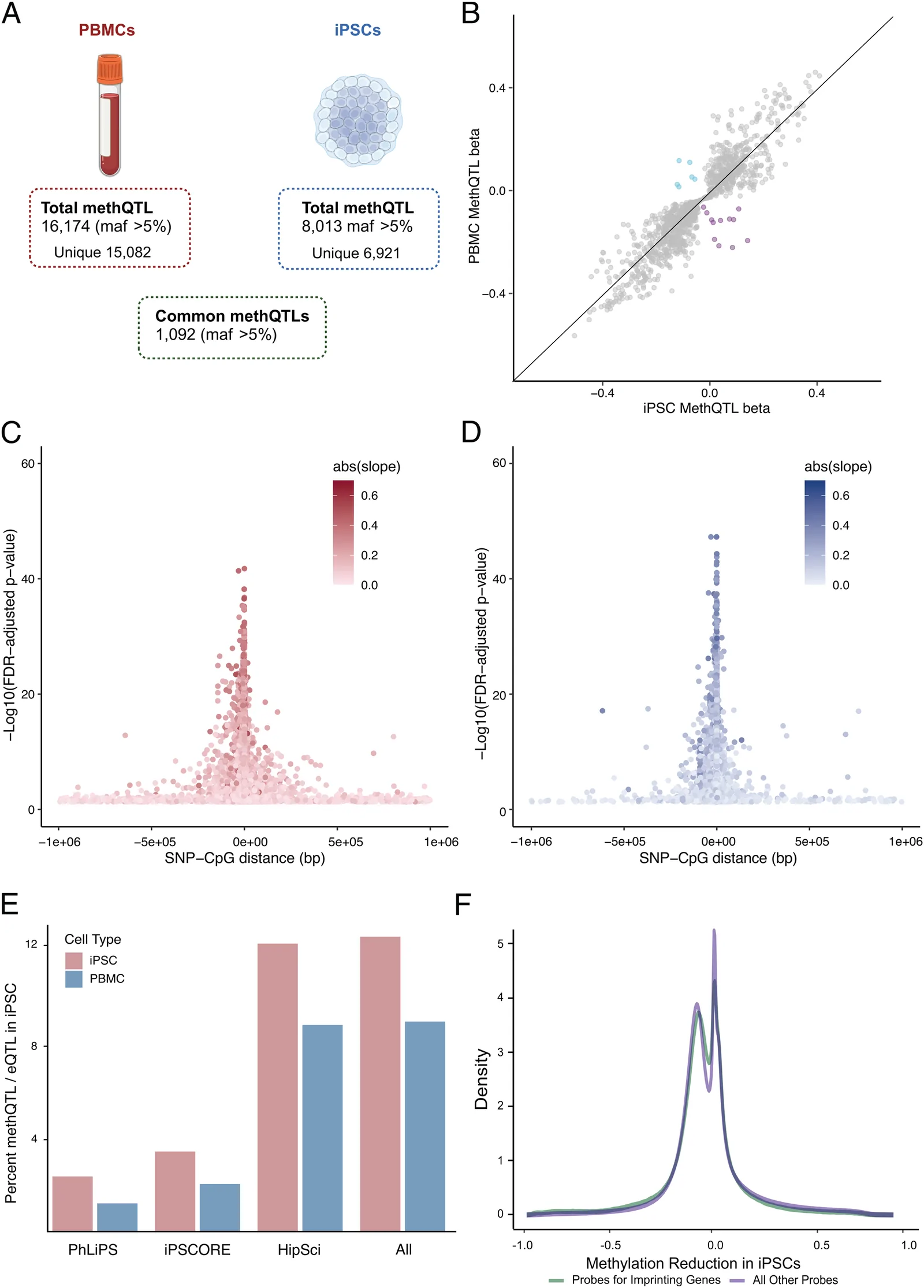
Characterization of methQTL in PBMCs and iPSCs. (A) Number of methQTL with maf > 5% identified by cell type (FDR corrected p-value < 0.05). (B) Scatter plot of beta values of significant methQTL shared across cell types, with iPSC values on the x-axis and PBMC values on the y-axis. Blue points represent CpG-SNP pairs in which the alternative allele of the SNP increases methylation in PBMCs but not iPSCs, and purple dots represent CpG-SNP pairs in which the alternative allele reduces the methylation of the CpG in PBMCs and not iPSCs. (C) The distribution p-values of CpG-SNP pairs in PBMCs (red) and (D) iPSCs (blue**)** with respect to the distance between features. Darker color indicates a stronger influence of genotype on methylation. **(**E) Percent of methQTL variants also identified as eQTL in published iPSC studies. **(**F) Density of the change in methylation in PBMCs upon reprogramming into iPSCs separated by probes for imprinting genes (green) and all other probes (purple).

The EWAS data show that donor age does not contribute to the overall CpG methylation signal in iPSCs. Given that these cells are cultured under consistent conditions, any exposure-related methylation signals will likely be equivalent across lines, leaving genetics and cellular differentiation state as the most likely drivers of CpG methylation across the genome. We, therefore, evaluated patterns of genetically correlated CpG levels by identifying methylation quantitative trait loci (methQTL) in iPSCs and their donor PBMCs. Using tensorQTL [33], we considered *cis*-methQTL to include variants + /-1 Mb window to the CpG site, and identified 16,174 methQTL in PBMCs at a minor allele frequency (maf) >5% (Fig 4A and S5 Table). As with the EWAS, we repeated methQTL analysis in PBMCs using cell type proportions as a covariate (S6 Table). Inclusion of cell type covariates resulted in a slight reduction in total methQTL identified [16],063), however almost 72% of sites overlapped between both analyses [13],448) regardless of the covariate inclusion. Including cell type covariates resulted in 2,615 new methQTL, and there were 2,726 distinct methQTL identified when cell type covariates were not used. In iPSC, we recovered 8,013 methQTL in at maf > 5% (Fig 4A and S7 Table). There were 1,092 methQTL that were statistically significant in both PBMCs and iPSCs. Examining these shared methQTL, we found that the effect sizes were generally consistent regarding direction and magnitude for both (Fig 4B and S8 Table). However, a subset of CpG sites with methQTL detected in both PBMCs and iPSCs had opposite directions of effect (Fig 4B). When comparing PBMC to iPSC, the majority of the significant methQTL (~90%) were only found in one of the two assayed cell states. The large difference in total methQTL numbers identified between cell types may be a result of the cell type heterogeneity of PBMCs, however without direct comparison to purified peripheral blood cell populations this is speculative.

We saw more significant associations, i.e., methQTL with lower p-values, were localized to within 0.5 Mb of the CpG probe with which they were associated in both PBMCs and iPSCs (Fig 4C and D). Notably, very few methQTL probes were shared with CpG sites within the epigenetic clocks used to estimate epigenetic age (S6A Fig), further demonstrating that methylation at specific genomic sites is independently influenced by age or genetic sequence. There was a significant correlation between the beta estimates for methQTL between PBMCs and iPSCs (Pearson correlation coefficient = 0.92), and the distribution of methQTL beta values was also similar between the different cell types (S6B and C Fig). These results suggest that methQTL have similar overall properties between PBMCs and iPSCs, and in a number of cases the same SNP-probe pairs are congruent in both cell types.

Next, we hypothesized that methQTL may also act as eQTL due to the role of DNA methylation in the regulation of transcription. To answer this, we intersected methQTL from each cell type with eQTL previously identified in three large scale iPSC studies [39–41]. We found intersection of methQTL in both cell types identifying 1,204 SNP-probe-gene sets in PBMCs and 850 SNP-probe-gene sets in iPSCs across all three datasets, however we show a significantly higher percentage of variants with QTL in both modalities in iPSCs (12.4% of iPSC methQTL) relative to PBMCs (8.8% of PBMC methQTL, χ^2^ = 63.97, df = 1, p < 0.00001) (Fig 4E; S9 Table). Knowing that genetic imprinting occurs in the germline, we wondered if genetic imprinting also occurs during iPSC reprogramming. To test this, first we calculated the change in methylation at each probe from the differentiated PBMCs to the undifferentiated iPSC state for each donor. Then plotted the density of the change in methylation for the subset of probes that are known to be imprinted in humans relative to the change in methylation of all other probes (Fig 4F). Based on this analysis there appears to be no enrichment in iPSC for methylation of probes associated with imprinting genes.

**Fig 5:**
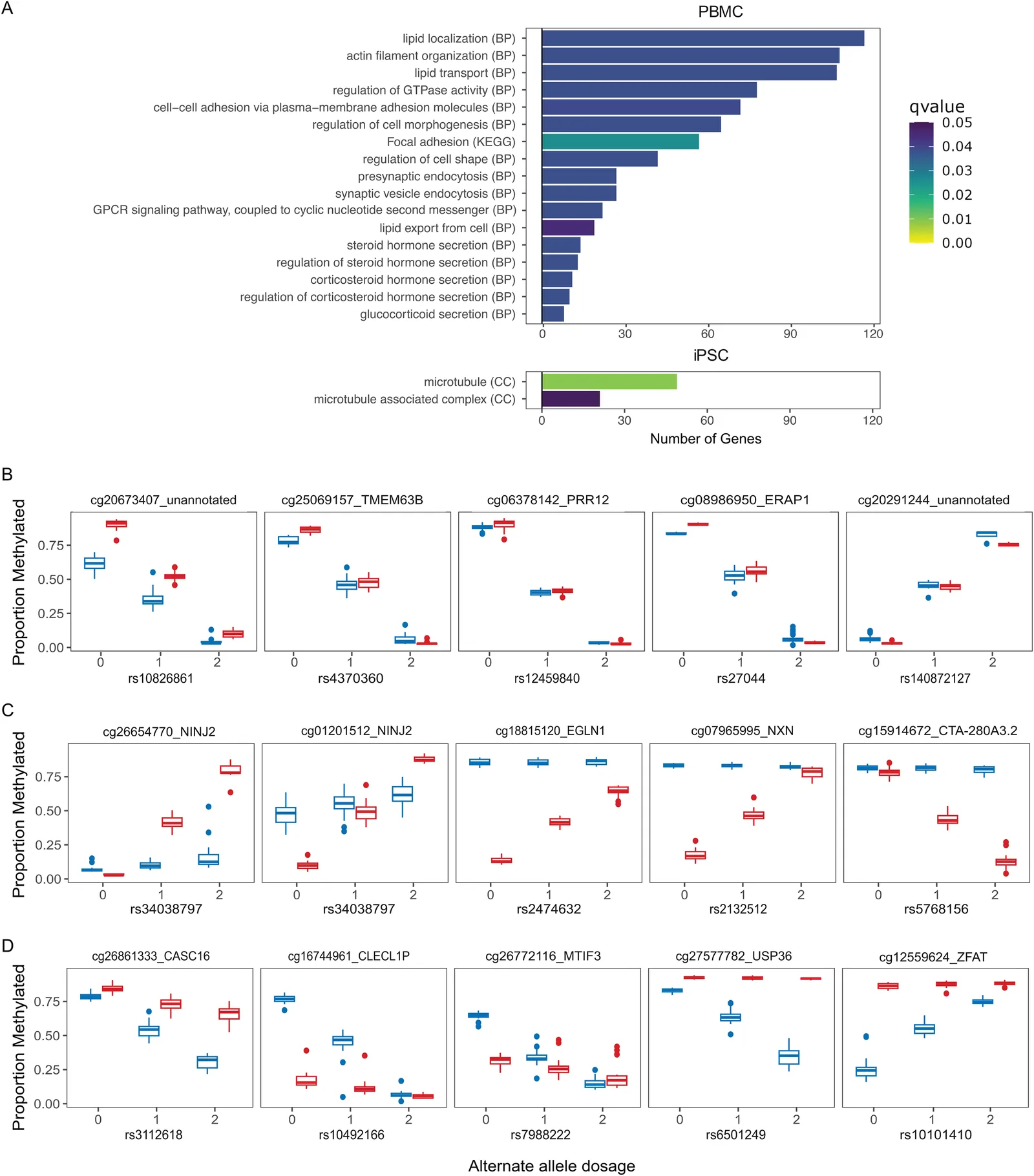
Cell type-specific methQTL enrichment. (A) Gene Ontology terms identified for PBMC-specific methQTL (upper panel) and iPSC-specific methQTL (lower panel). Only biological process (BP) terms were found to be significantly associated with PBMCs, and only cellular component (CC) terms were associated with iPSCs. (B) Examples of SNP-probe pairs with the same significant effect in both cell types, (C) SNP-probe pairs that were only significant in PBMCs, and (D) SNP-probe pairs that were only significant in iPSC. PBMC values are red and iPSC values are blue.

Querying annotated genes for methylation probes, methQTL signals that were significant only in PBMCs were enriched for genes that are involved in lipid localization and transport, and pathways associated with steroid secretion, while methQTL signals that were significant only in iPSCs are enriched for genes that are involved in microtubules and microtubule-associated processes (Fig 5A). These analyses reinforce that despite methQTL having similar overall properties, the complement of methQTL that can be detected in each cell type are specific and related to cell function. To identify examples of the relationships that methQTL have between cell types, we examined the genotype:methylation relationship for probes near genes in either iPSCs or PBMCs. We identified a set of probes that shows consistent methQTL across both cell types with the same direction of effect in both cell types (Fig 5B). The gene annotations for these probes (*TMEM63B*, *PRR12*, *ERAP1*, and two unannotated) identified genes that have low tissue specificity and are moderately expressed across many tissues.

Next, we examined a set of methQTL SNP-probe pairs that are significant in PBMCs but not iPSCs (Fig 5C) which included a SNP-probe pair associated with EGL9 Family hypoxia-inducible factor 1 (*EGLN1*). The cg18815120 site was fully methylated in iPSCs but showed genotype specific methylation in PBMCs. Interestingly, mutations in *EGLN1* are linked to two different autosomal dominant blood disorders, familial erythrocytosis-3 and high altitude adaptation hemoglobin, reflecting an important role specific to blood cells [42,43]. We identified a final set of SNP-probe pairs that are significant in iPSCs but not PBMCs (Fig 5D). The cg27577782 probe showed full methylation across genotypes in PBMCs but genotype specific changes in methylation in iPSCs. This site is associated with Ubiquitin-Specific Protease 36 (*USP36*) a gene that displays broad deubiquitinase activity that is linked to numerous biological processes including selective autophagy and ribosome biosynthesis [44]. These examples suggest that the cell type specificity of some methQTL may contribute to complex phenotypes at the organismal level.

**Fig 6:**
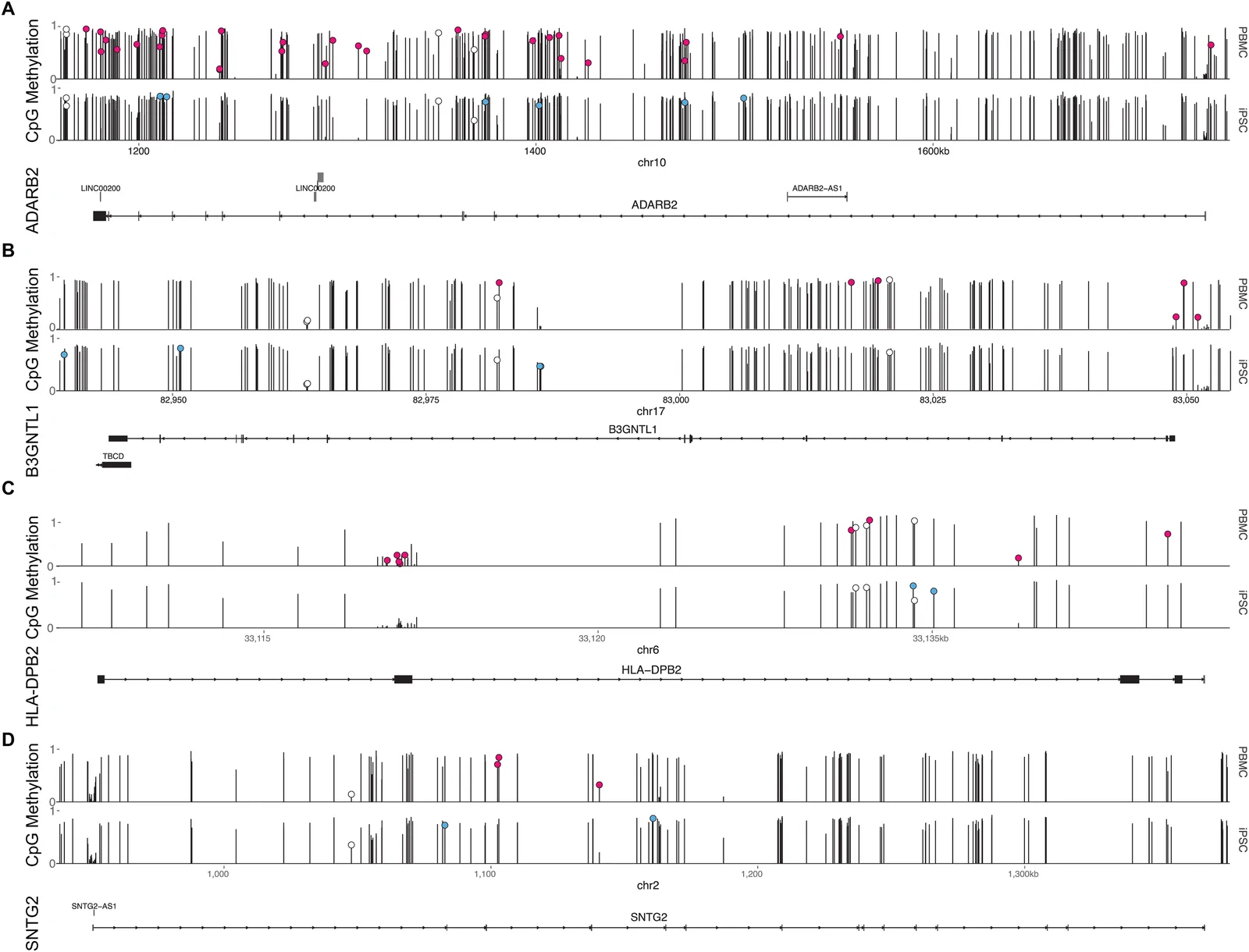
Genomic loci where multiple methylation signals can be detected but methQTL show cell type specificity. (A) *ADARB2* locus plot shows methylation probes across the region with average methylation values on the vertical axis for PBMCs (upper plots) and iPSCs (lower plots). Methylation probes significantly associated with a neighboring genotype locus (methQTL) are labeled with circles, and are either PBMC-specific (red), iPSC-specific (blue), or present in both cell types (white). (B) *B3GNTL1* locus plot*,* (C) *HLA-DPB2* locus plot, and (D) *SNTG2* locus plot. Ideograms below each pair of methylation tracks show gene coordinates for which the locus is named.

When cataloging enrichments, we noted that there was a greater proportional overlap in listed genes near methylation probes for which there was a methQTL than for methQTL overall. This suggested that, for any given locus, there might be multiple methQTL signals of which a proportion would be specific to a given cell type, extending the single examples discussed above. To evaluate this possibility, we identified loci for which there were more than ten methQTL with signals in either iPSCs and/or PBMCs that were annotated to the same unique gene. For all candidate loci (~200), we plotted the mean methylation level in all samples for each cell type and annotated those which showed a significant methQTL signal in either iPSCs or PBMCs. We found that such loci have multiple methylation signals and that, as expected, a subset of those methylation signals were also methQTL, some of which were shared between cell types, but with a greater extent of cell-type specificity (Fig 6). For example, at the *ADARB2*/*ADARB2-AS1* locus on chr10, which has been previously shown to be differentially methylated in Alzheimer’s disease [45], we identified methQTL-associated methylation in iPSCs that was fully unmethylated in PBMCs and vice-versa (Fig 6A). We show additional examples near *B3GNTL1*, *HLA-DPB2*, and *SNTG2* (Fig 6B-D). These results further support the concept that detection of a methQTL in a given cell type is facile when the underlying methylation feature shows intermediate methylation state in the cell population.

## Conclusions

In this study, we set out to examine the effects of genetic variation in iPSCs relative to the PBMCs they were derived from on DNA methylation by measuring methQTL signals across the genome. We had the expectation that iPSCs are immature cells and hence lack age-related methylation changes, giving us the ability to isolate methQTL independent of aging. However, comparing PBMC methylation from the same donors, we find that methQTL are most strongly influenced by cell differentiation status.

To support these analyses, we generated a set of iPSCs from donors across human lifespan. Similar population-level collections have been reported in the past and have proven useful for genome-wide studies of human genetics and genomics at the endogenous level [46–48]. Our collection is characterized by the inclusion of donors without any clinical diagnoses at the time of collection and across a wide range of ages. We also included individuals with diverse ancestries; thus, this population partially reflects the community composition from which donors were recruited. Importantly for the current study, the same series of donors consented to genetic analysis, including genotyping and methylation from the PBMCs used to generate iPSCs, allowing for direct comparison between these two cell types.

Using DNA methylation, we confirm that iPSCs are epigenetically young compared to PBMCs from the same donors in regions where strong correlations between chronological age and methylation status were readily identified. This underlying result was stable across multiple clocks representing different sets of methyl-CpG sites across the genome [11,26–28]. Additionally, we used EWAS to evaluate age associations with methylation across the genome and noted that while there were many robust methylation signals in PBMCs, there were only a small number of nominally significant correlations in iPSCs. Several observations suggest that these candidate sites are likely not to be robust. First, given that we used a false discovery rate correction of 5%, the observed number of candidates (four) is lower than expected by chance sampling of ~900,000 candidate sites. Second, estimated p-values for these four sites were only marginally above the significance threshold compared to the sites in PBMCs that greatly exceeded the same threshold. Third, examination of specific candidate CpG sites suggests that these are highly methylated in PBMCs and that any nominal correlation may be driven by a subset of iPSC lines with incomplete CpG methylation. Overall, these results demonstrate that iPSCs do not reflect the donor’s age from which they are derived, at least at the DNA epigenetic modification level.

Given that we have shown that iPSC generation removes the correlated effects of age on DNA methylation, and that the iPSC lines are maintained in a consistent environment, we therefore set out to examine genetic influences on CpG methylation by enumerating methQTL across the genome in both iPSC and PBMCs from the same donors. We find that methQTL share many characteristics between these two cell types, including an enrichment around gene bodies and proximity to the transcriptional start sites of genes similar to previous studies of methQTL in multiple tissues [5]. Despite these general similarities in where methQTL are found across the genome, the actual sites that show methQTL are generally distinct between PBMCs and iPSCs. Specifically, while we identified ~16,000 and ~8,000 methQTL in PBMCs and iPSCs, respectively, only ~1000 were shared between the two cell types, despite being derived from the same donors. Thus, while methQTL in different cell types may share similar generic properties, they are found in different genomic regions.

Examining which genes are associated with methQTL restricted to either cell type, we found that there were enrichments in PBMCs that reflect the function of these cells in the immune system. While the role of specific CpGs in regulating lipid localization in PBMCs or microtubules in iPSCs is speculative, additional follow-up and CRISPR-based functional studies in both iPSCs and differentiated cells could be a good next step in the validation of these results. Furthermore, at many loci there were specific CpG methylation events that show genetic influence in only one cell type where intermediate methylation status was noted, i.e., that the methylation probe was neither fully methylated or fully unmethylated. Based on this data, we infer that DNA methylation is influenced by both cell type and genetic variation so that the two together affect measurable methQTL patterns. Specifically, we propose that for a methQTL to be measurable in a given differentiated cell type, the chromatin in that region must be permissive for transcription. A relationship between CpG methylation and changes in chromatin accessibility has been identified across species and tissues [49], although it is not always clear whether methylation drives chromatin changes or vice versa. Because the current analysis focuses on genetic variation, we can reasonably conclude that methQTL are driven by genetic variants in each cell type but that the differences in methQTL between differentiation states are likely to be consequential to the establishment of cell identity, driven by cell type-specific transcriptional program and hence chromatin accessibility patterns. Future studies may take iPSCs and differentiate them into different target cell types, which could test this inference.

We acknowledge that there are several limitations to this study. First, while we could identify methQTL, our sample size is relatively small and would benefit from increased numbers to recover methQTL of smaller effect sizes. A power analysis suggests that the current data are powered to detect QTL with moderate or large effect sizes. At a 80% power threshold, the minimum detectable effect thresholds are 29.27% for iPSC and 30.87% for PBMCs. Where 93.67% of the methQTL identified in iPSC and 99.97% of those in PBMCs exceed these computed moderate effect size thresholds. In order to detect methQTL of smaller effect size would require a larger sample size or a meta-*cis*-methQTL analysis with published datasets for iPSCs and PBMCs (or whole blood as proxy) QTL datasets for DNA methylation. Second, we compared our iPSC data to PBMCs, which are heterogeneous cell populations and may underestimate the effects restricted to cellular subtypes. Cell type proportions for PBMCs were estimated using methylation patterns and included as a covariate in the analysis, however the heterogeny of cell types and methylation within those different cell types could explain some of the differences we see between PBMC and iPSC. While unlikely due to reduction in power, it is possible that the mixed cell types may be capturing multiple methylation signals in PBMCs, which could inflate the total number of methQTL identified relative to the more homogenous iPSCs. Additional confirmation in more restricted cell types would be helpful to determine the role of cell type heterogeneity in methQTL detection. However, this seems to be an unlikely confound, as we detected greater numbers of methQTL with biological enrichments in immune-related genes in the PBMCs than in the iPSCs that we would expect to be more homogenous in terms of cell type. This also does not change our main message that iPSCs lose their age-related methylation upon reprogramming, while age-related signals are detected in PBMCs. Finally, this analysis is restricted to only the CpG sites included and passing filters on the Human Methylation EPIC chip and to SNPs with maf > 5% on the Neuro Global Diversity Array with Neuro Booster Content. While these arrays cover a large part of the human genome, it is possible that there may be some methQTL that are missed due to lack of coverage in one or both assays. Long-read DNA sequencing may be a useful method to probe these missing regions for methQTL in future studies.

Overall, the current study attempted to disambiguate aging and cell type on CpG methylation state as defined by genetic variation captured in iPSCs. In contrast to our initial expectation, we find that methQTL are influenced strongly by cell type in that PBMCs could identify many more methQTL and many unique methQTL in functionally relevant gene enrichments compared to iPSCs. These results imply that methQTL detection may depend on cell type to a greater extent than has previously been appreciated.

## Supporting information

S1 Fig iPSC lines are generated from a cohort of healthy donors. (A) Schematic diagram of the workflow used for iPSCs generation. PBMC samples were reprogrammed using CytoTune-iPS 2.0 Sendai Reprogramming Kit. Two clones per donor were generated, and a PluriTest characterization was applied to each clone. The PluriTest characterization assay includes 1) pluripotency testing; 2) karyotyping; and 3) mycoplasma testing. (B) The Pluritest results are represented in a pluripotency plot. The x-axis shows the novelty score results, while the y-axis represents the pluripotency score results per each clone. The red and blue background depicts the empirical distribution of the pluripotent (red) and non-pluripotent (blue) samples in the reference data set of hESCs, iPSCs, and somatic cell tissues. The tested iPSC samples are black points. A non-iPSC sample derived from HELA cells (yellow) was included as a negative control for non-pluripotency.
(PNG)

S2 Fig Correlation between clones and between donors. Methylation variability between clone-to-clone and PBMC-to-clone comparison. Pearson correlation coefficient comparisons between iPSC clones from the same donor (white), same donor PBMC to iPSC clones (gray), and unrelated donors (including both clone to clone and PBMC to clone comparison). The group comparison is on the x-axis, while the y-axis represents the Pearson correlation coefficients per group. All group comparisons were significantly different from each other (ANOVA: d*f* = 4, *F* = 10320, *p* < 2 × 10^−16^; Tukey HSD, all comparisons: *p* < 2 × 10^−16^).
(PNG)

S3 Fig Correlation matrix between PBMCs and iPSC clones. Pearson correlation analysis was performed using 775 informative methylation probes. Informative methylation probes were selected by intersecting the top 5000 most variable probes for iPSCs and PBMCs.
(PNG)

S4 Fig EWAS in PBMCs adjusted for cell type proportions. Manhattan plot showing significant associations between DNA methylation at common CpG sites and age in cell type proportion adjusted PBMCs.
(PNG)

S5 Fig Telomere length in iPSC. Correlation between donor age at the time of collection (x-axis) and Telomere Restriction Fragment TRF length (y-axis) in iPSC. Telomere length is not correlated to biological age at collection in iPSCs, r^2^=−0.0065.
(PNG)

S6 Fig Overlap of epigenetic aging clock and methQTL CpG methylation sites. (A) For each of the clocks listed on the horizontal axis, the proportion of methylation sites that were identified as significant methQTLs in iPSCs (blue), PBMCs (red), both cell types (black), or not significant in either cell type (gray). (B) beta distribution of the significant CpG-SNP pairs identified in PBMCs. (C) Beta distribution of the significant CpG-SNP pairs identified in iPSCs.
(PNG)

S1 Table Summary of demographics of donor samples for the initial cohort. (XLSX)

S2 Table PluriTest results for iPSC clones. (XLSX)

S3 Table Epigenetic age versus chronological age. (XLSX)

S4 Table Predicted PBMC cell type proportions. (XLSX)

S5 Table Intersection of significant EWAS hits with probes in included Epigenetic clocks. (XLSX)

S6 Table Significant methQTL identified in PBMCs. (XLSX)

S7 Table Significant methQTL identified in PBMCs when estimating cell type proportions. (XLSX)

S8 Table Significant methQTL identified in iPSCs. (XLSX)

S9 Table Shared methQTL between PMBCs and iPSCs. (XLSX)

S10 Table MethQTL overlapping with eQTL in previously published studies. (XLSX)

## References

[1] Law JA, Jacobsen SE. Establishing, maintaining and modifying DNA methylation patterns in plants and animals. Nat Rev Genet. 2010;11(3):204–20. doi: 10.1038/nrg2719 20142834 PMC3034103

[2] D’Aquila P, Rose G, Bellizzi D, Passarino G. Epigenetics and aging. Maturitas. 2013;74(2):130–6. doi: 10.1016/j.maturitas.2012.11.005 23245587

[3] Ferrucci L, Gonzalez-Freire M, Fabbri E, Simonsick E, Tanaka T, Moore Z, et al. Measuring biological aging in humans: A quest. Aging Cell. 2020;19(2):e13080. doi: 10.1111/acel.13080 31833194 PMC6996955

[4] Marioni RE, Suderman M, Chen BH, Horvath S, Bandinelli S, Morris T, et al. Tracking the epigenetic clock across the human life course: a meta-analysis of longitudinal cohort data. J Gerontol A Biol Sci Med Sci. 2019;74(1):57–61. doi: 10.1093/gerona/gly060 29718110 PMC6298183

[5] Gibbs JR, van der Brug MP, Hernandez DG, Traynor BJ, Nalls MA, Lai SL, et al. Abundant quantitative trait loci exist for DNA methylation and gene expression in human brain. PLoS Genet. 2010;6(5):e1000952. doi: 10.1371/journal.pgen.1000952 20485568 PMC2869317

[6] Nabais MF, Gadd DA, Hannon E, Mill J, McRae AF, Wray NR. An overview of DNA methylation-derived trait score methods and applications. Genome Biol. 2023;24(1):28. doi: 10.1186/s13059-023-02855-7 36797751 PMC9936670

[7] Villicaña S, Bell JT. Genetic impacts on DNA methylation: research findings and future perspectives. Genome Biol. 2021;22(1):127. doi: 10.1186/s13059-021-02347-6 33931130 PMC8086086

[8] Zhang D, Cheng L, Badner JA, Chen C, Chen Q, Luo W, et al. Genetic control of individual differences in gene-specific methylation in human brain. Am J Hum Genet. 2010;86(3):411–9. doi: 10.1016/j.ajhg.2010.02.005 20215007 PMC2833385

[9] Cuomo ASE, Seaton DD, McCarthy DJ, Martinez I, Bonder MJ, Garcia-Bernardo J, et al. Single-cell RNA-sequencing of differentiating iPS cells reveals dynamic genetic effects on gene expression. Nat Commun. 2020;11(1):810. doi: 10.1038/s41467-020-14457-z 32041960 PMC7010688

[10] Salles J, Eddiry S, Amri S, Galindo M, Lacassagne E, George S, et al. Differential DNA methylation in iPSC-derived dopaminergic neurons: a step forward on the role of SNORD116 microdeletion in the pathophysiology of addictive behavior in Prader-Willi syndrome. Mol Psychiatry. 2024;29(9):2742–52. doi: 10.1038/s41380-024-02542-4 38561465

[11] Horvath S. DNA methylation age of human tissues and cell types. Genome Biol. 2013;14(10):R115. doi: 10.1186/gb-2013-14-10-r115 24138928 PMC4015143

[12] Lo Sardo V, Ferguson W, Erikson GA, Topol EJ, Baldwin KK, Torkamani A. Influence of donor age on induced pluripotent stem cells. Nat Biotechnol. 2017;35(1):69–74. doi: 10.1038/nbt.3749 27941802 PMC5505172

[13] Miller JD, Ganat YM, Kishinevsky S, Bowman RL, Liu B, Tu EY, et al. Human iPSC-based modeling of late-onset disease via progerin-induced aging. Cell Stem Cell. 2013;13(6):691–705. doi: 10.1016/j.stem.2013.11.006 24315443 PMC4153390

[14] Ruiz S, Diep D, Gore A, Panopoulos AD, Montserrat N, Plongthongkum N. Identification of a specific reprogramming-associated epigenetic signature in human induced pluripotent stem cells. Proc Natl Acad Sci. 2012;109(40):16196–201. doi: 10.1073/pnas.1202352109 22991473 PMC3479609

[15] Roy R, Kuo PL, Candia J, Sarantopoulou D, Ubaida-Mohien C, Hernandez D, et al. Epigenetic signature of human immune aging in the GESTALT study. eLife. 2023;12:e86136. doi: 10.7554/eLife.86136 37589453 PMC10506794

[16] Roy R, Ramamoorthy S, Shapiro BD, Kaileh M, Hernandez D, Sarantopoulou D, et al. DNA methylation signatures reveal that distinct combinations of transcription factors specify human immune cell epigenetic identity. Immunity. 2021;54(11):2465–80.e5. doi: 10.1016/j.immuni.2021.10.001 34706222 PMC9190145

[17] Tsitsipatis D, Martindale JL, Ubaida-Mohien C, Lyashkov A, Yanai H, Kashyap A, et al. Proteomes of primary skin fibroblasts from healthy individuals reveal altered cell responses across the life span. Aging Cell. 2022;21(5):e13609. doi: 10.1111/acel.13609 35429111 PMC9124301

[18] Tsitsipatis D, Martindale JL, Mazan-Mamczarz K, Herman AB, Piao Y, Banskota N, et al. Transcriptomes of human primary skin fibroblasts of healthy individuals reveal age-associated mRNAs and long noncoding RNAs. Aging Cell. 2023;22(11):e13915. doi: 10.1111/acel.13915 37462262 PMC10652340

[19] Tumasian RA 3rd, Harish A, Kundu G, Yang J-H, Ubaida-Mohien C, Gonzalez-Freire M, et al. Skeletal muscle transcriptome in healthy aging. Nat Commun. 2021;12(1):2014. doi: 10.1038/s41467-021-22168-2 33795677 PMC8016876

[20] Bandres-Ciga S, Faghri F, Majounie E, Koretsky MJ, Kim J, Levine KS, et al. NeuroBooster Array: A Genome-Wide Genotyping Platform to Study Neurological Disorders Across Diverse Populations. Mov Disord. 2024;39(11):2039–48. doi: 10.1002/mds.29902 39283294 PMC11568947

[21] Sun Q, Liu W, Rosen JD, Huang L, Pace RG, Dang H, et al. Leveraging TOPMed imputation server and constructing a cohort-specific imputation reference panel to enhance genotype imputation among cystic fibrosis patients. HGG Adv. 2022;3(2):100090. doi: 10.1016/j.xhgg.2022.100090 35128485 PMC8804187

[22] Min JL, Hemani G, Davey Smith G, Relton C, Suderman M. Meffil: efficient normalization and analysis of very large DNA methylation datasets. Bioinformatics. 2018;34(23):3983–9. doi: 10.1093/bioinformatics/bty476 29931280 PMC6247925

[23] Chen Y, Lemire M, Choufani S, Butcher DT, Grafodatskaya D, Zanke BW, et al. Discovery of cross-reactive probes and polymorphic CpGs in the Illumina Infinium HumanMethylation450 microarray. Epigenetics. 2013;8(2):203–9. doi: 10.4161/epi.23470 23314698 PMC3592906

[24] McCartney DL, Walker RM, Morris SW, McIntosh AM, Porteous DJ, Evans KL. Identification of polymorphic and off-target probe binding sites on the Illumina Infinium MethylationEPIC BeadChip. Genom Data. 2016;9:22–4. doi: 10.1016/j.gdata.2016.05.012 27330998 PMC4909830

[25] Pidsley R, Zotenko E, Peters TJ, Lawrence MG, Risbridger GP, Molloy P, et al. Critical evaluation of the Illumina MethylationEPIC BeadChip microarray for whole-genome DNA methylation profiling. Genome Biol. 2016;17(1):208. doi: 10.1186/s13059-016-1066-1 27717381 PMC5055731

[26] Hannum G, Guinney J, Zhao L, Zhang L, Hughes G, Sadda S, et al. Genome-wide methylation profiles reveal quantitative views of human aging rates. Mol Cell. 2013;49(2):359–67. doi: 10.1016/j.molcel.2012.10.016 23177740 PMC3780611

[27] Levine ME, Lu AT, Quach A, Chen BH, Assimes TL, Bandinelli S, et al. An epigenetic biomarker of aging for lifespan and healthspan. Aging (Albany NY). 2018;10(4):573–91. doi: 10.18632/aging.101414 29676998 PMC5940111

[28] Zhang Q, Vallerga CL, Walker RM, Lin T, Henders AK, Montgomery GW, et al. Improved precision of epigenetic clock estimates across tissues and its implication for biological ageing. Genome Med. 2019;11(1):54. doi: 10.1186/s13073-019-0667-1 31443728 PMC6708158

[29] Pelegí-Sisó D, de Prado P, Ronkainen J, Bustamante M, González JR. methylclock: a Bioconductor package to estimate DNA methylation age. Bioinformatics. 2021;37(12):1759–60. doi: 10.1093/bioinformatics/btaa825 32960939

[30] Livak KJ, Schmittgen TD. Analysis of Relative Gene Expression Data Using Real-Time Quantitative PCR and the 2−ΔΔCT Method. Methods. 2001;25(4):402–8. doi: 10.1006/meth.2001.1262 11846609

[31] Bakulski KM, Feinberg JI, Andrews SV, Yang J, Brown S, L McKenney S, et al. DNA methylation of cord blood cell types: Applications for mixed cell birth studies. Epigenetics. 2016;11(5):354–62. doi: 10.1080/15592294.2016.1161875 27019159 PMC4889293

[32] Manichaikul A, Mychaleckyj JC, Rich SS, Daly K, Sale M, Chen W-M. Robust relationship inference in genome-wide association studies. Bioinformatics. 2010;26(22):2867–73. doi: 10.1093/bioinformatics/btq559 20926424 PMC3025716

[33] Taylor-Weiner A, Aguet F, Haradhvala NJ, Gosai S, Anand S, Kim J, et al. Scaling computational genomics to millions of individuals with GPUs. Genome Biol. 2019;20(1):228. doi: 10.1186/s13059-019-1836-7 31675989 PMC6823959

[34] Yu G, Wang L-G, Han Y, He Q-Y. clusterProfiler: an R package for comparing biological themes among gene clusters. OMICS. 2012;16(5):284–7. doi: 10.1089/omi.2011.0118 22455463 PMC3339379

[35] Xu S, Hu E, Cai Y, Xie Z, Luo X, Zhan L, et al. Using clusterProfiler to characterize multiomics data. Nat Protoc. 2024;19(11):3292–320. doi: 10.1038/s41596-024-01020-z 39019974

[36] Bock C, Kiskinis E, Verstappen G, Gu H, Boulting G, Smith ZD, et al. Reference Maps of human ES and iPS cell variation enable high-throughput characterization of pluripotent cell lines. Cell. 2011;144(3):439–52. doi: 10.1016/j.cell.2010.12.032 21295703 PMC3063454

[37] Huang J, Wang F, Okuka M, Liu N, Ji G, Ye X, et al. Association of telomere length with authentic pluripotency of ES/iPS cells. Cell Res. 2011;21(5):779–92. doi: 10.1038/cr.2011.16 21283131 PMC3203670

[38] Takasawa K, Arai Y, Yamazaki-Inoue M, Toyoda M, Akutsu H, Umezawa A, et al. DNA hypermethylation enhanced telomerase reverse transcriptase expression in human-induced pluripotent stem cells. Hum Cell. 2018;31(1):78–86. doi: 10.1007/s13577-017-0190-x 29103143

[39] Kilpinen H, Goncalves A, Leha A, Afzal V, Alasoo K, Ashford S, et al. Common genetic variation drives molecular heterogeneity in human iPSCs. Nature. 2017;546(7658):370–5. doi: 10.1038/nature22403 28489815 PMC5524171

[40] Panopoulos AD, Smith EN, Arias AD, Shepard PJ, Hishida Y, Modesto V, et al. Aberrant DNA Methylation in Human iPSCs Associates with MYC-Binding Motifs in a Clone-Specific Manner Independent of Genetics. Cell Stem Cell. 2017;20(4):505–17.e6. doi: 10.1016/j.stem.2017.03.010 28388429 PMC5444384

[41] Pashos EE, Park Y, Wang X, Raghavan A, Yang W, Abbey D, et al. Large, Diverse Population Cohorts of hiPSCs and Derived Hepatocyte-like Cells Reveal Functional Genetic Variation at Blood Lipid-Associated Loci. Cell Stem Cell. 2017;20(4):558–70.e10. doi: 10.1016/j.stem.2017.03.017 28388432 PMC5476422

[42] Lorenzo FR, Huff C, Myllymäki M, Olenchock B, Swierczek S, Tashi T, et al. A genetic mechanism for Tibetan high-altitude adaptation. Nat Genet. 2014;46(9):951–6. doi: 10.1038/ng.3067 25129147 PMC4473257

[43] Percy MJ, Zhao Q, Flores A, Harrison C, Lappin TRJ, Maxwell PH, et al. A family with erythrocytosis establishes a role for prolyl hydroxylase domain protein 2 in oxygen homeostasis. Proc Natl Acad Sci U S A. 2006;103(3):654–9. doi: 10.1073/pnas.0508423103 16407130 PMC1334658

[44] Shao L, Guo M, Kou Q, Guo Y, Li X, Li F. The role of USP36 in ribosome biogenesis and other pathophysiological processes. Front Mol Biosci. 2025;12:1650908. doi: 10.3389/fmolb.2025.1650908 40909126 PMC12404947

[45] Konki M, Malonzo M, Karlsson IK, Lindgren N, Ghimire B, Smolander J, et al. Peripheral blood DNA methylation differences in twin pairs discordant for Alzheimer’s disease. Clin Epigenetics. 2019;11(1):130. doi: 10.1186/s13148-019-0729-7 31477183 PMC6721173

[46] Bonder MJ, Smail C, Gloudemans MJ, Frésard L, Jakubosky D, D’Antonio M, et al. Identification of rare and common regulatory variants in pluripotent cells using population-scale transcriptomics. Nat Genet. 2021;53(3):313–21. doi: 10.1038/s41588-021-00800-7 33664507 PMC7944648

[47] Bressan E, Reed X, Bansal V, Hutchins E, Cobb MM, Webb MG, et al. The Foundational Data Initiative for Parkinson Disease: Enabling efficient translation from genetic maps to mechanism. Cell Genom. 2023;3(3):100261. doi: 10.1016/j.xgen.2023.100261 36950378 PMC10025424

[48] Schwartzentruber J, Foskolou S, Kilpinen H, Rodrigues J, Alasoo K, Knights AJ, et al. Molecular and functional variation in iPSC-derived sensory neurons. Nat Genet. 2018;50(1):54–61. doi: 10.1038/s41588-017-0005-8 29229984 PMC5742539

[49] Li S, Peng Y, Panchenko AR. DNA methylation: Precise modulation of chromatin structure and dynamics. Curr Opin Struct Biol. 2022;75:102430. doi: 10.1016/j.sbi.2022.102430 35914496

